# Short-term health effects of tear agents chlorobenzylidenemalononitrile and oleoresin capsicum during the civil riots of Santiago de Chile in 2019–2020

**DOI:** 10.1007/s43188-025-00282-3

**Published:** 2025-02-27

**Authors:** Carlos Jara Bravo, José Antonio Cernuda Martínez, Pedro Arcos González

**Affiliations:** Unit for Research in Emergency and Disasters, Faculty of Medicine, University of Oviedo, Campus del Cristo, 33006 Oviedo, Spain

**Keywords:** Tear gas, Health effects, Riots

## Abstract

Chlorobenzylidenemalononitrile (CS) and Oleoresin Capsicum (OC) were tear gasses used as anti-riot control agents during social unrest riots in Chile (October 2019–March 2020). This study posed as a research question what were the short-term health effects of CS and OC and their patterns of temporal occurrence in a sample of inhabitants and health care volunteer brigades of the Plaza Italia (city of Santiago, Chile) during the riots. A retrospective cross-sectional study was conducted in 112 exposed people (inhabitants and health care volunteers) affected by CS and OC. 62 harmful effects were studied classified in three time periods of occurrence: immediate effects (between exposure and one hour), secondary effects (from one hour after exposure and up to 24 h), and subsequent effects (days after exposure). The use of CS and OC in Santiago riots 2019–2020 produced harmful effects on both groups: inhabitants and brigade health care volunteers. The frequency of effects was, from most to least common: 62.5% eye pain or burning, 56.2% throat irritation, 54.4% respiratory distress, 52.6% skin pain or burning, 51.7% impaired vision, 37.5% skin erythema, 31.2% headache, 31.2% irregular breathing, 25.8% conjunctival injection, 29.4% nausea, 27.6% disorientation, 26.7 high blood pressure, 25.8% lip pain, 24.1% rhinitis, 24.1% skin sensitivity, 22.3% diarrhea, 20.5% contact dermatitis, 18.7% conjunctivitis, 16.9% skin vesicles, 16% tachycardia, 14.4 cough with phlegm and 9.8% corneal abrasion. 22 effects were more frequent (*p* < 0.05) in health care volunteers than among residents. High blood pressure was more frequent (*p* < 0.05) among residents. Immediate most frequent effects were pain or burning, impaired vision, respiratory difficulty, irregular breathing, skin pain and burning, skin erythema, nausea, tachycardia, and hypertension. Secondary effects were diarrhea, skin vesicles, and eye pain or burning. Subsequent most frequent effects among healthcare personnel were conjunctivitis, skin pain, burning, rhinitis, and diarrhea. Among residents, the most common effects were skin pain, burning, and impaired vision.

## Introduction

Tear agents are chemical compounds that temporarily incapacitate people by causing irritation to the eyes, mouth, throat, lungs, and skin. Some of them are used as anti-riot agents such as chlorobenzylidenemalononitrile, chloroacetophenone, chloropicrin, oleoresincapsicum, bromobenzylcyanide, and dibenzoxazepine. They can be in liquid or fine dust form that is released into the air as fine droplets or particles and people are exposed to them through contact with the skin, eyes, or breathing. The extent of its adverse effects depends on the amount of agent to which a person was exposed, the location of exposure (indoor versus outdoor), the method of use, and the duration of exposure. The most common health effects are usually short-term and non-permanent, but there can also be serious effects including death [1, 2].

The 1993 International Chemical Weapons Convention qualifies tear gas as anti-riot agents and prohibits their use against enemy troops in times of war, but allows its use against the civilian population in times of peace [3]. The use of tear gas to control civil disturbances is difficult to carry out adequately and serious traumatic injuries from the explosion of tear gas bombs, as well as lethal toxic injuries, have been documented [4, 5].

Between October 2019 and March 2020, Chile experienced a series of riots and demonstrations triggered by the increase in public transport fares in the Metropolitan Region that led to demonstrations throughout almost the entire country demanding greater social equality, recognition, and the guarantee of social and economic rights [6]. There were violent actions and crimes that seriously affected public and private infrastructure, such as the burning of stations and trains of the Santiago metro [7].

On October 18, the Chilean Government decreed a state of emergency in the Metropolitan Region, and on the 19th, a state of emergency and curfew in Santiago, Valparaíso, and the Province of Concepción, later extended to most regions of the country. On October 25, 1.2 million people participated in a demonstration in Santiago, and on November 14, political parties reached an agreement to begin a process to change the Constitution, including an initial referendum in April 2020 [8].

In the Metropolitan Region, the area with the most demonstrations was Plaza Italia, whose surroundings were transformed into a conflict scenario called Zone Zero [9, 10]. To control the demonstrations, the authorities used lethal and non-lethal elements and devices, among the latter large quantities of tear gas, generating a high number of affected people. This period was characterized by high levels of violence against protesters by authorities and excessive use of force [11].

In the Plaza Italia sector, due to the large number of people injured and affected by violence, the individual efforts of civil society were coordinated to provide first aid care through the constitution of the so-called Health Brigades of the Plaza de la Dignidad. [12], composed of volunteers (doctors, nurses, etc.) equipped with health supplies, radio communications, and decontamination resources who met on demonstration days at health posts to care for the injured.

On the other hand, Plaza Italia and its surroundings is a mainly residential sector, with homes, office buildings, shops, Metro stations, large parks, and squares. Since October 18, 2019, this area and its residents and regular visitors were continuously affected by the demonstrations until March 2020. Residents were affected by tear gas, directly by passing through contaminated sectors, and also indirectly once the demonstrations were over, as they resided in a sector saturated with chemical substances.

The need for the study is due to the fact that residents or regular visitors would be directly and indirectly exposed to tear gas, but no information was available on the health effects related to their exposure. The objective of this study is to describe the short-term health effects produced by the tear gasses Chlorobenzylidenemalononitrile (CS) and Oleoresin Capsicum (OC) in the inhabitants and brigade health care volunteers of the Plaza Italia sector of the city of Santiago, Chile during the riots and its temporal pattern of occurrence.

## Materials and methods

A retrospective observational study was carried out in Santiago de Chile between the months of February and March 2022 in a study population of 112 people affected by the tear agents CS and OC in the context of the riots in the Plaza Italia sector. A *probable case of poisoning* was defined as a case with compatible clinical signs in which there is a high index of suspicion due to the presence of a credible threat or history of the case concerning the location and time of exposure to a tear agent [13]. The participants in the study were classified into two groups: recurring visitors to the area composed of health care volunteers members of the health brigades (n = 49), and residents or inhabitants in the Plaza Italia sector (n = 63). Regarding the recruiting system used, the survey was given to the brigade leaders and they offered each of the brigade members the option of answering them using Google Forms. As for the residents of the area, the surveys were provided to the leaders of the neighborhood association in the area and they provided the information to their lists of neighbors.

There was no precise information on how many health brigade members attended the riots because no official registration was made, but our reports indicate an estimate number of 80. Regarding residents, the Plaza de Italia is mostly a commercial area and very few people normally living there, around 300 people. There is no precise estimate available of how many of the area's usual inhabitants remained residing in the area during the riots because the level of violence caused many residents to temporarily leave for a few days or weeks to another location.

The existing literature on the potentially harmful health effects of CS and OC agents was reviewed and a list was prepared in which the effects were grouped into ocular, respiratory, skin, gastrointestinal, neurological, cardiac, reproductive, and fatigue.[14–20]. The complete list of the 62 variables studied can be seen in Annex I. Information was collected through a questionnaire prepared ad hoc and distributed online using the Google Forms application. A total of 250 surveys were distributed to residents of the area, and an additional 70 were distributed to healthcare volunteers.

For each harmful effect collected in the questionnaire, three response options were included relative to the relative moment in which they were perceived by the participants: immediate effects (observed between exposure and one hour), secondary effects (from one hour after exposure and up to 24 h) and subsequent effects (days after exposure).

The collected information was transferred to an Excel spreadsheet to later be analyzed. Absolute and relative frequencies were used in the analysis. After verifying the normality of the distribution of the variables, the association between them was confirmed using the Chi-square test with Yates' correction. Even considering the different characteristics of the two groups compared, we decided including the Chi-squared frequency comparison test compared to the option of not doing any type of test. When a difference is indicated to be statistically significant, *p* < 0.05 is assumed. Stata v.15 software was used in the statistical analysis. The study was reviewed and accepted by the University's ethics committee.

## Results

A number of 112 people answered the questionnaire. Of them, 63 (56.2%) were residents of the area and 49 (43.7%) were health care volunteers of the aid brigades. The response rate was 25.2% for residents of the area and 70% for volunteers.

Of the residents, 67.2% were women, 31.3% were men, and 1.5% was non-binary. Of the healthcare volunteers, 54.2% were women and 45.8% were men. 78.1% of the residents did not have any previous health problems and neither did 91.7% of the brigade members.

The frequency of harmful health effects in the 112 individuals studied was, from most to least common: 62.5% eye pain or burning, 56.2% throat irritation, 54.4% respiratory distress, 52.6% skin pain or burning, 51.7% impaired vision, 37.5% skin erythema, 31.2% headache, 31.2% irregular breathing, 25.8% conjunctival injection, 29.4% nausea, 27.6% disorientation, 26.7 high blood pressure, 25.8% lip pain, 24.1% rhinitis, 24.1% skin sensitivity, 22.3% diarrhea, 20.5% contact dermatitis, 18.7% conjunctivitis, 16.9% skin vesicles, 16% tachycardia, 14.4 cough with phlegm and 9.8% corneal abrasion.

**Fig. 1:**
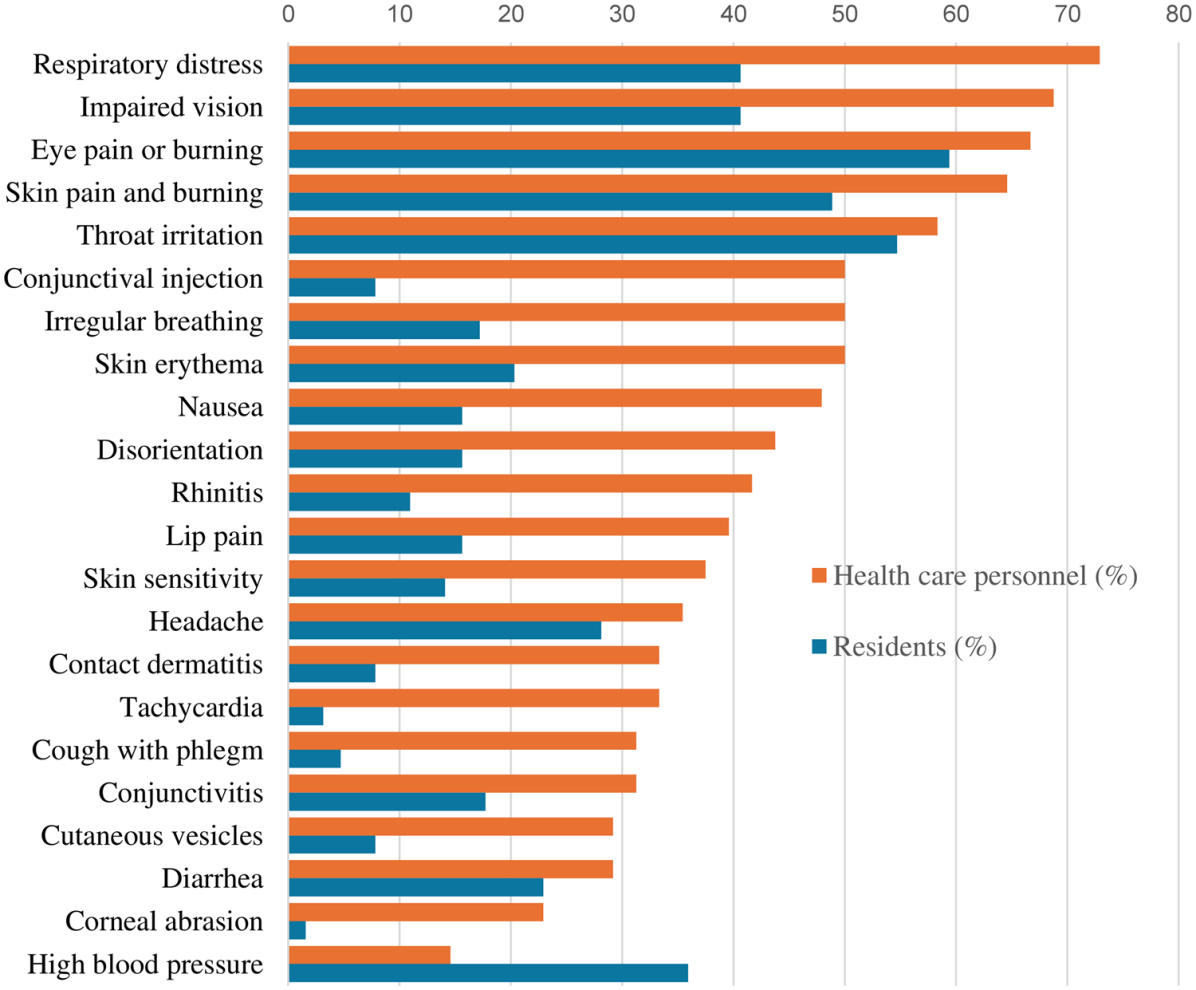
Percentage of residents and healthcare personnel who suffered harmful effects

Table 1 shows the comparative frequency of those specific harmful effects for which there was a significant difference in the occurrence between the group of healthcare volunteers and residents. The percentage of healthcare volunteers and residents suffering harmful effects at any time (immediate + secondary + subsequent) is shown in Fig. 1. Twenty-two harmful effects were significantly more frequent (*p* < 0.05) in the group of healthcare volunteers than among residents. Only high blood pressure was significantly more frequent among residents than among healthcare volunteers. The most frequent effects in healthcare volunteers were respiratory distress (72.9%), impaired vision (68.7%), eye pain or burning (66.6%9 and skin pain or burning (64.5). Among residents, the two most frequent effects were eye pain or burning (59.3%), throat irritation (54.6%), skin pain and burning (48.8), and high blood pressure (35.9%).

**Table 1:** Frequency of harmful effects on health personnel and residents according to the time of occurrence (immediate, secondary and subsequent)

Effect	Time of occurrence	Resident n (%)	Healthcare personnel n (%)	χ^2^	*p* value
Eye pain or burning	Immediate	31 (48.44)	26 (54.17)	16.96	0.005
	Immediate and secondary	3 (4.69)	10 (20.83)		
	Immediate, secondary and subsequent	2 (3.13)	3 (6.25)		
	Subsequent effects	1 (1.56)	0		
	Secondary effects	6 (9.38)	6 (12.50)		
Impaired vision	Immediate effects	11 (17,19)	29 (60.42)	29.88	< 0.000
	Immediate and secondary	1(1.56)	4 (8.33)		
	Immediate, secondary and subsequent	0	0		
	Subsequent effects	5 (7.81)	0		
	Secondary effects	10 (15.63)	4 (8.33)		
Conjunctival injection	Immediate effects	3 (4.69)	18 (37.50)	49.31	< 0.000
	Immediate, secondary and subsequent	0	1 (1.56)		
	Immediate and secondary	1 (1.56)	10 (20.83)		
	Subsequent effects	0	2 (4.17)		
	Secondary effects	2 (3.13)	4 (8.33)		
Conjunctivitis	Immediate effects	4 (6.25)	6 (12.50)	19.65	0.001
	Immediate and secondary	0	4 (8.33)		
	Immediate, secondary and subsequent	0	1 (2.08)		
	Subsequent effects	2 (3.13)	4 (8.33)		
	Secondary effects	0	5 (10.42)		
Corneal abrasion	Immediate effects	0	6 (12.5)	16.96	0.002
	Immediate and secondary	1 (1.56)	1 (2.08)		
	Immediate, secondary and subsequent	0	0		
	Subsequent effects	1 (1.56)	0		
	Secondary effects	0	5 (10.42)		
Throat irritation	Immediate effects	31 (48.44)	23 (47.92)	17.95	0.003
	Immediate and secondary	4 (6.25)	10 (20.83)		
	Immediate, secondary and subsequent	1 (1.56)	5 (10.42)		
	Subsequent effects	0	1 (2.08)		
	Secondary effects	4 (6.25)	4 (8.33)		
Respiratory distress	Immediate effects	19 (29.69)	35 (72.92)	24.49	< 0.000
	Immediate and secondary	4 (6.25)	3 (6.25)		
	Immediate, secondary and subsequent	2 (3.13)	2 (4.17)		
	Subsequent effects	0	0		
	Secondary effects	7 (10.94)	0		
Irregular breathing	Immediate effects	7 (10.94)	24 (50.00)	27.86	< 0.000
	Immediate and secondary	1 (1.56)	3 (6.25)		
	Immediate, secondary and subsequent	2 (3.13)	3 (6.25)		
	Subsequent effects	2 (3.13)	0		
	Secondary effects	2 (3.13)	0		
Rhinitis	Immediate effects	3 (4.69)	13(27.08)	34.59	< 0.000
	Immediate and secondary	2 (3.13)	7 (14.58)		
	Immediate, secondary and subsequent	3 (4.69)	7 (14.58)		
	Subsequent effects	1 (1.56)	3 (6.25)		
	Secondary effects	3 (4.69)	4 (8.33)		
Cough with phlegm	Immediate effects	0	11 (22.92)	30.99	< 0.000
	Immediate and secondary	1 (1.56)	1 (2.08)		
	Immediate, secondary and subsequent	0	6 (12.50)		
	Subsequent effects	3 (4.69)	2 (4.17)		
	Secondary effects	0	2 (4.17)		
Skin pain and burning	Immediate effects	25 (39.06)	26 (54.17)	24.62	< 0.000
	Immediate and secondary	0	1 (2.08)		
	Immediate, secondary and subsequent	4 (6.25)	13 (27.08)		
	Subsequent effects	5 (7.81)	4 (8.33)		
	Secondary effects	2	1 (2.08)		
Skin erythema	Immediate effects	12 (18.75)	20 (41.67)	52.51	< 0.000
	Immediate and secondary	1 (1.56)	14 (29.17)		
	Immediate, secondary and subsequent	1 (1.56)	4 (8.33)		
	Subsequent effects	0	0		
	Secondary effects	1 (1.56)	4 (8.33)		
Contact dermatitis	Immediate effects	3 (4.69)	12 (25.00)	32.22	< 0.000
	Immediate and secondary	0	7 (14.58)		
	Immediate, secondary and subsequent	3 (4.69)	5 (10.42)		
	Subsequent effects	1 (1.56)	1 (2.08)		
	Secondary effects	2 (3.13)	4 (8.33)		
Cutaneous vesicles	Immediate effects	3 (4.69)	6 (12.5)	13.69	0.033
	Immediate and secondary	1 (1.56)	0		
	Immediate, secondary and subsequent	2 (3.13)	0		
	Subsequent effects	0	2 (4.17)		
	Secondary effects	1 (1.56)	3 (6.25)		
Skin sensitivity	Immediate effects	7 (10.94)	13 (27.08)	31.69	< 0.000
	Immediate and secondary	2 (3.13)	9 (18.75)		
	Immediate, secondary and subsequent	5 (7.81)	9 (18.75)		
	Subsequent effects	2 (3.13)	1 (2.08)		
	Secondary effects	0	4 (8.33)		
Lip pain	Immediate effects	7 (10.94)	17 (35.42)	29.44	< 0.000
	Immediate and secondary	0	8 (16.67)		
	Immediate, secondary and subsequent	0	1 (2.08)		
	Subsequent effects	2 (3.13)	0		
	Secondary effects	1 (1.56)	2 (4.17)		
Nausea	Immediate effects	7 (10.94)	20 (41.67)	26.34	< 0.000
	Immediate and secondary	1 (1.56)	6 (12.50)		
	Immediate, secondary and subsequent	0	1 (2.08)		
	Subsequent effects	1 (1.56)	1 (2.08)		
	Secondary effects	2 (3.13)	2 (4.17)		
Diarrhea	Immediate effects	11 (17,19)	1 (2.08)	22.05	0.001
	Immediate and secondary	3 (4.69)	1 (2.08)		
	Immediate, secondary and subsequent	5 (7.81)	3 (6.25)		
	Subsequent effects	1 (1.56)	2 (4.17)		
	Secondary effects	0	11 (22.92)		
Headache	Immediate effects	13 (20,31)	14 (29.17)	23.09	0.001
	Immediate and secondary	2 (3.13)	10 (20.83)		
	Immediate, secondary and subsequent	3 (4.69)	3 (6.25)		
	Subsequent effects	0	1 (2.08)		
	Secondary effects	5 (7.81)	2 (4.17)		
Disorientation	Immediate effects	8 (12.50)	20 (41.67)	15.39	0.004
	Immediate and secondary	1 (1.56)	1 (2.08)		
	Immediate, secondary and subsequent	0	0		
	Subsequent effects	0	1 (2.08)		
	Secondary effects	2 (3.13)	0		
Tachycardia	Immediate effects	2 (3.13)	16 (33,33)	23.31	< 0.000
	Immediate and secondary	1 (1.56)	3 (6.25)		
	Immediate, secondary and subsequent	0	1 (2.08)		
	Subsequent effects	0	0		
	Secondary effects	0	0		
High blood pressure	Immediate effects	21 (32.81)	7 (14.58)	17.27	0.008
	Immediate and secondary	3 (4.69)	1 (2.08)		
	Immediate, secondary and subsequent	9 (14.06)	1 (2.08)		
	Subsequent effects	1 (1.56)	0		
	Secondary effects	1 (1.56)	0

**Fig. 2:**
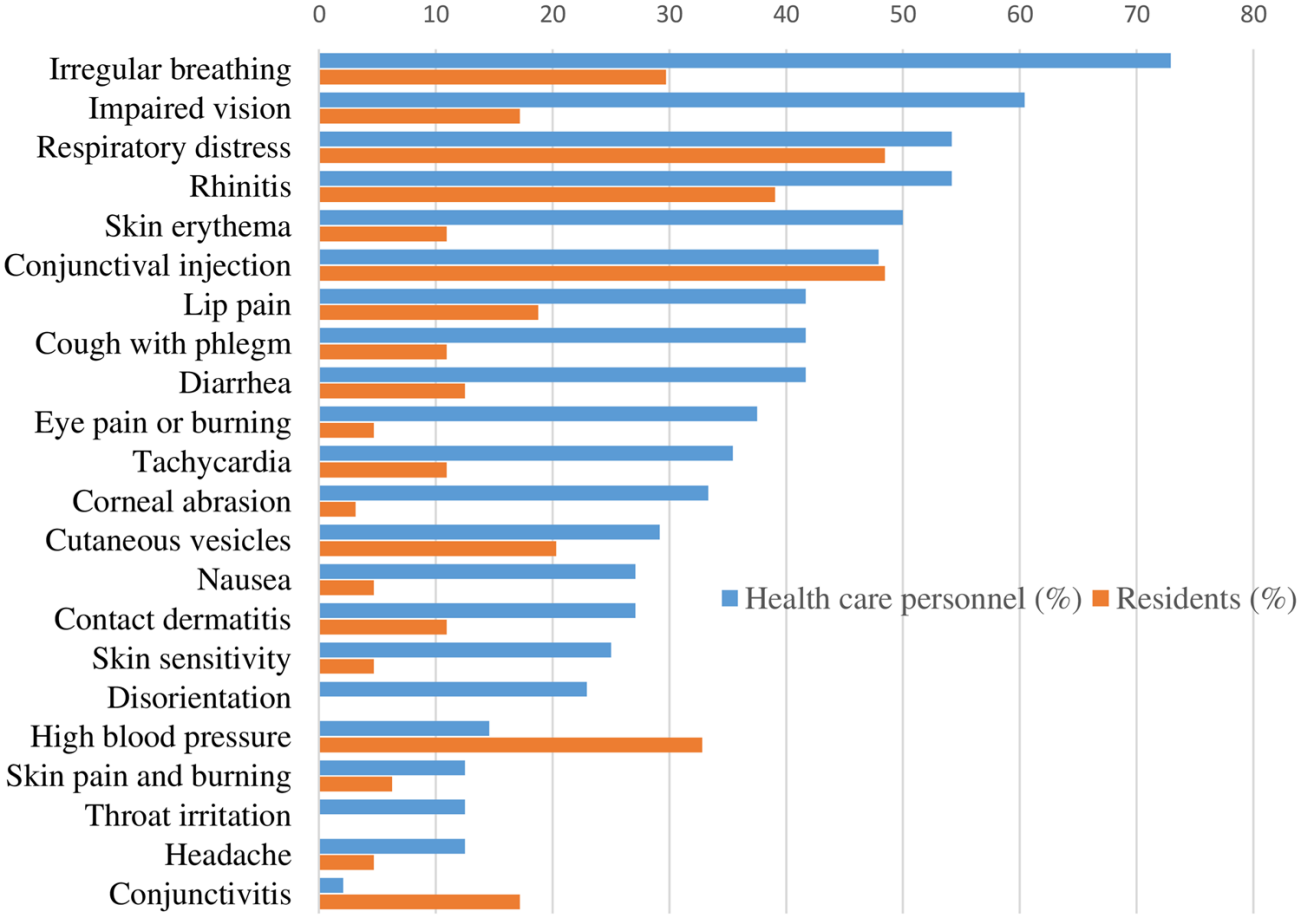
Percentage of residents and healthcare personnel who suffered harmful immediate effects (within the first hour after exposure)

Figure 2 shows the frequency of effects during the first hour after exposure (immediate effects). At the ocular level, pain or burning and impaired vision affected brigade members significantly more than residents. Respiratory effects, respiratory distress, and irregular breathing were also significantly more frequent among brigade members than among residents. At the skin level, the differences between both groups were in the presence of pain and burning and skin erythema. There was also a significantly higher frequency of nausea among brigade members than among residents. Regarding cardiocirculatory effects in the hour post-exposure, both tachycardia and hypertension were significantly more frequent among brigade members than among residents.

**Fig. 3:**
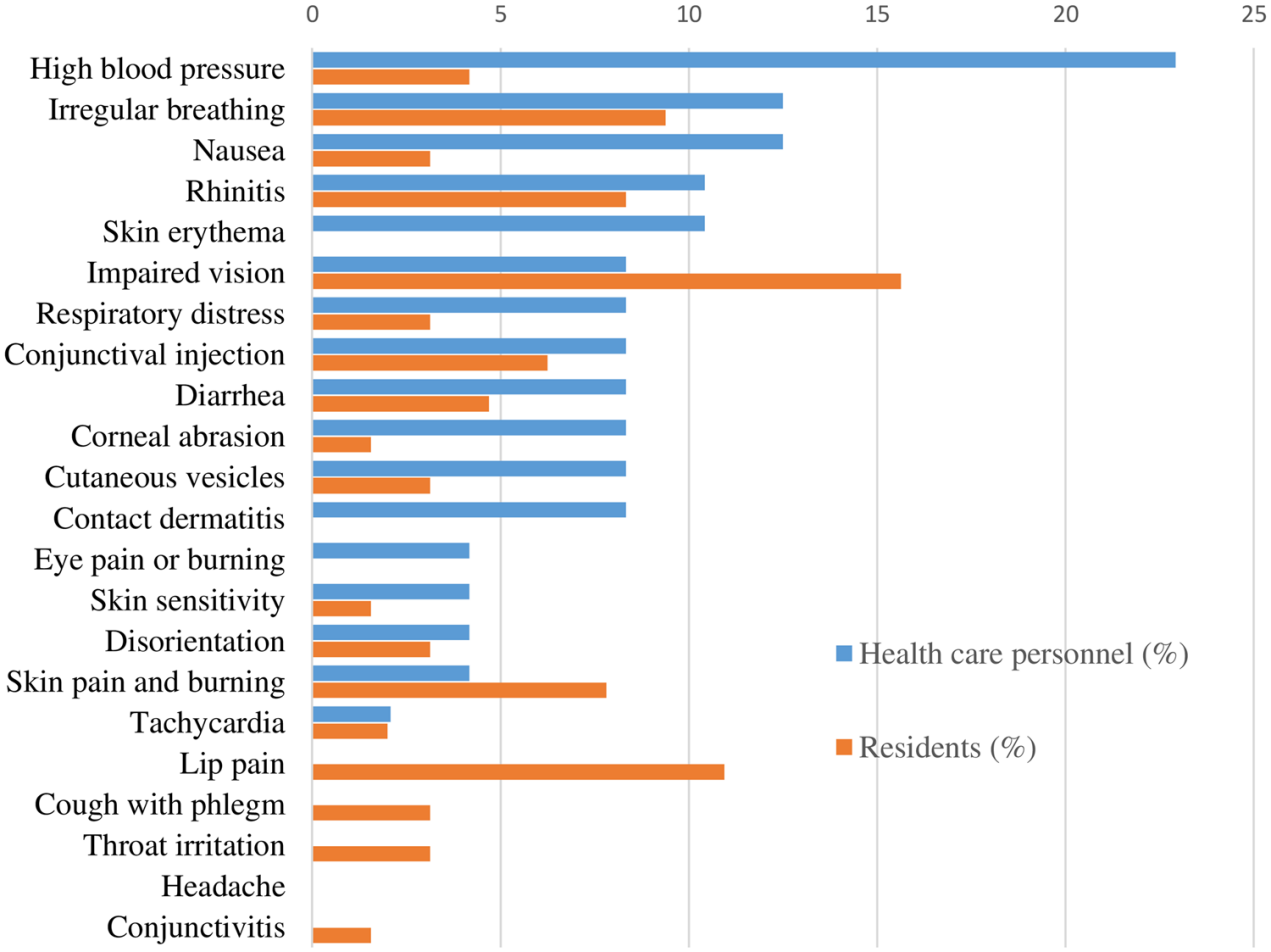
Percentage of residents and healthcare personnel who suffered harmful secondary effects (after the first hour after exposure and before 24 h)

**Fig. 4:**
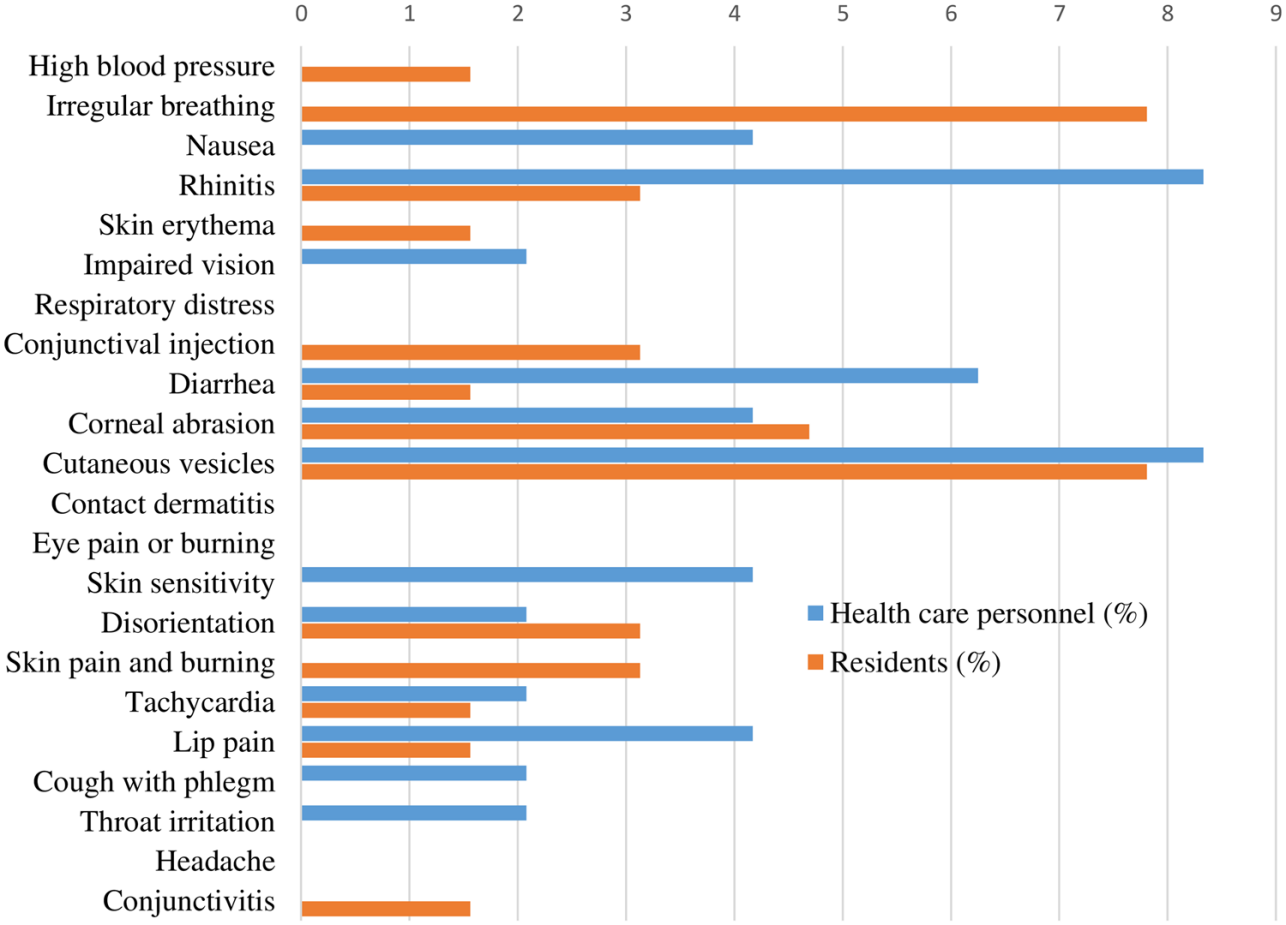
Percentage of exposed people with subsequent harmful effects (after 24 h and up to several days after exposure) in residents and healthcare personnel

The frequency of secondary effects (from one hour after exposure to 24 h later) is shown in Fig. 3. Diarrhea, skin vesicles, and eye pain or burning were the significantly more frequent effects among brigade members compared to residents. Among residents, the most common was impaired vision. Figure 4 shows the frequency of subsequent effects (between 24 h and several days after exposure). The most frequent effects among brigade members were conjunctivitis, skin pain, burning, rhinitis, and diarrhea. Among residents, skin pain burning and impaired vision were reported.

## Discussion

CS and OC gases are two widely used chemical agents for riot control causing irritation and discomfort, sharing some common features but distinct chemical compositions and effects. CS gas is an organic compound derived from benzylidenemalononitrile, a member of the lachrymatory agents family, used in the form of an aerosol or as a solid in riot control canisters that release the substance into the air. CS is slightly soluble in water but highly soluble in organic solvents like ethanol or acetone. CS gas primarily works by stimulating the sensory nerves in the eyes, skin, and mucous membranes (especially the respiratory system). When inhaled or contacted, it causes intense irritation of the eyes, nose, throat, and respiratory tract. The symptoms of exposure include eyes intense burning, excessive tearing, blepharospasm and blurred vision, skin redness, irritation, itching, and a burning sensation, coughing, choking, shortness of breath, chest tightness, and potentially wheezing, disorientation, panic, nausea, and headaches. Symptoms usually last 15–30 min, though the discomfort can persist longer, especially if exposure is prolonged. Chronic exposure can lead to lung damage, especially for individuals with pre-existing respiratory conditions. In rare cases, exposure to large quantities or prolonged exposure could lead to permanent damage to the eyes or respiratory system. High concentrations in confined spaces can cause severe respiratory distress and even death, although fatalities are rare.

OC Gas is an oily extract from the fruit of hot chili peppers, particularly from varieties like “Capsicum annuum” and it is usually found in liquid form in pepper spray canisters and can be dispersed as a fine mist, gel, or foam. OC is soluble in oil but not in water and works by irritating the transient receptor potential vanilloid 1, which are responsible for detecting heat and pain. It induces inflammation in the mucous membranes, especially the eyes and airways. Symptoms of exposure include intense burning, watering, swollen eyelids, and temporary blindness, skin severe burning sensation, redness, swelling, and irritation, coughing, choking, difficulty breathing, and a feeling of suffocation, panic, disorientation, and intense discomfort. Some people may experience nausea, dizziness, or vomiting. Burning sensation and discomfort can last from 20 min to an hour, but symptoms can persist longer depending on the amount of exposure.

OC short-term Risks include temporary vision impairment (inability to see for several minutes) and eye pain, respiratory distress, especially for individuals with asthma or other lung diseases, severe discomfort and psychological effects, including panic or anxiety. OC long-term risks include eye damage (prolonged or intense exposure may cause corneal damage or other eye problems), respiratory effects (prolonged exposure or repeated use could exacerbate chronic respiratory conditions like asthma, bronchitis, or emphysema), and skin damage (intense exposure can cause burns or allergic reactions)”.

The immediate or short-term effects of tear gases are usually more noticeable and are due to irritation caused by the chemicals in the gas. They are usually temporary and disappear after moving away from the area of exposure or after rinsing the eyes and face with water. Long-term effects can be more severe and persist after exposure has ended.

People exposed to tear gases may experience symptoms immediately after exposure. The majority are benign with their irritant effects starting within 30 min [21] such as ocular, cutaneous, nasal, digestive and pulmonary [22, 23]. Serious exposures can produce more severe injuries to the eye, skin, and respiratory tract requiring intensive care. But there may also be later effects [24, 25], for example, chest pain can develop at a later moment [26], as well as psychological problems [27].

When exposure occurs in closed places and at high doses of the agent, depending on the dose–response relationship, there may be serious effects such as blindness, glaucoma, and even death due to severe chemical burns to the throat and lungs or due to respiratory failure. Also, nausea, vomiting, and diarrhea have been described if high concentrations are attained, such as when exposure occurs in a confined space or when a long duration of exposure occurs [20].

Long-term effects such as eye problems or respiratory problems [28] and permanent disabling injuries have also been described as a result of the use of these agents in more than 1% of exposed people [29]. Exceptional effects of the type of severe multisystem disease due to hypersensitivity rather than direct tissue toxicity as above have also been described [30].

The frequency of harmful effects depends on the agent, dose used, and conditions of exposure. A systematic review by Haarr et al. In 2017 [31] reported a frequency of 8.7% of serious effects in all body systems that require medical treatment, 17% of moderate effects, and 74.3% of minor effects. CS used to be safe when used at low concentrations (1 part per 100,000,000) as a microparticulate cloud for riot control purposes [32] but experimental studies have found that ocular damage occurs after the application of high concentrations of CS to the eye, especially when applied in solution [33]. There have also been case reports of significant ophthalmological sequelae [34].

This frequency of minor effects coincides with that of our study where the majority of injuries affected the eyes and skin of those exposed with averages of 80% and 74%, respectively. All the effects studied in our study were significantly more frequent in the group of brigade members than in the residents, except high blood pressure, which was more frequent in the latter. This could be due to the different age composition of both groups since the resident group had a significantly higher average age.

The harmful health effects of tear gas have been documented and evidence demonstrates that they have the potential to cause serious harm and present specific threats to vulnerable populations, including children, women, and individuals affected by respiratory, cutaneous, and cardiovascular morbidities [14]

Despite this, the fact that they are generally considered non-lethal and the limited duration of most of the effects, most of them lasting less than two weeks except in some cases with previous pathologies such as asthma [35], may lead one to think that they are relatively harmless, which is not true [36–39]. Therefore, more research is needed to elucidate which conditions of use should be avoided and a serious reevaluation of chemical safety and more comprehensive exposure follow-up studies are necessary [15, 40]. From the toxicological point of view, we need more epidemiological and laboratory research to know the health consequences of exposure to full tear gas compounds such as CS. The possibility of health consequences in the long term, such as cancer, reproductive effects, and lung disease is particularly worrying given the multiple exposures suffered by demonstrators and non-demonstrators too in some areas of civil unrest [41–44].

This study has several limitations: the first one is that the harmful effects collected were “self-perceived” by the participants and not diagnosed by a medical professional. The second one is that obtaining an adequate sample in emergency situations with violence is difficult because the participating population in consecutive riots was not stable, unknown and numbers changed every day. It is also not possible to obtain a census of participants from which to obtain a representative sample. So, the sample only collects the information from the people who answered the questionnaire. The third one is that in the context in which the study was conducted (a riot area with high violence and a population that is very mobile from one area to another) it was impossible to analyse whether each participant was in an area with a certain concentration of tear gas dispersion.

As a conclusion, Chlorobenzylidenemalononitrile and Oleoresin Capsicumtear gas during social unrest in the Plaza de Italia area of Santiago, Chile between October 2019 and March 2020 produced health harmful effects on both the residents of the area and the brigade volunteers who provided health care for those affected. More than half of the exposed people studied presented eye pain or burning, throat irritation, respiratory difficulty, skin pain or burning, and impaired vision. And more than one in three had skin erythema, headache, and irregular breathing. Twenty two of the negative effects were significantly more frequent in the group of brigade members than among the residents and only high blood pressure was significantly more frequent among the residents.

In the first hour after exposure (immediate effects), the most frequent effects were pain or burning, impaired vision, respiratory difficulty, irregular breathing, skin pain and burning, skin erythema, nausea, tachycardia, and hypertension, all of which were significantly more frequent among the brigade members than among the residents. Between one hour after exposure and 24 h later, the most frequent effects were diarrhea, skin vesicles, and eye pain or burning, all of which were significantly more frequent among brigade members than among residents. Between 24 h and several days after exposure, the most frequent effects among brigade members were conjunctivitis, skin pain, burning, rhinitis, and diarrhea. Among residents, they were skin pain, burning, and impaired vision.

## Data Availability

Data available on request
